# Trauma and Social Pathways to Psychosis, and Where the Two Paths Meet

**DOI:** 10.3389/fpsyt.2021.804971

**Published:** 2022-01-10

**Authors:** Charles Heriot-Maitland, Til Wykes, Emmanuelle Peters

**Affiliations:** ^1^Department of Psychology, King's College London, Institute of Psychiatry, Psychology and Neuroscience, London, United Kingdom; ^2^Institute of Health and Wellbeing, Mental Health Research Facility, University of Glasgow, Glasgow, United Kingdom; ^3^South London and Maudsley NHS Foundation Trust, London, United Kingdom

**Keywords:** psychosis, trauma, shame, dissociation, attachment

## Abstract

The pathways from trauma—*via* dissociation—to psychosis have been thoroughly tested and evidenced, but what has received less attention has been the social pathways—*via* dissociation—to psychosis. Often social factors are more commonly linked to other influences, e.g., to appraisals and the creation of negative schema in cognitive models, or to unsupportive caregiving experiences where there is high “expressed emotion.” However, evidence is now emerging that negative social rank experiences, such as being excluded or shamed, may themselves have dissociative properties, which poses intriguing questions as to how trauma pathways and social pathways might interact. This article reviews the state of knowledge in trauma and social pathways to psychosis and then considers the potential mechanisms and the relationships between them, specifically (i) dissociation, (ii) attachment, and (iii) social rank. Recommendations are suggested for future modeling and testing of three-way interactions (dissociation × attachment × social rank) in the pathway from trauma to psychosis.

## Trauma/Adversity Pathways To Psychosis

### Empirical and Theoretical Associations of Trauma and Psychosis

The strength of the empirical evidence linking trauma and psychosis is reported to be of a magnitude comparable to the causal association between smoking and lung cancer (1). In a meta-analyses of 41 studies, Varese et al. (2) found that childhood adversity and trauma increases the odds of psychosis by 2.8, and that this increased risk of psychosis held across multiple different types of trauma (odds ratios for: sexual abuse 2.38, physical abuse 2.95, emotional abuse 3.40, bullying 2.39, and neglect 2.90). The only specific trauma type not significantly associated with psychosis risk was parental death (odds ratio 1.70). Importantly, there is also evidence of a cumulative relationship between trauma and psychosis, i.e., the more trauma types experienced, the greater likelihood of psychosis; for example, Shevlin et al. (3) reported that experiencing three types of trauma made the odds of psychosis 18 times more likely, and experiencing five types of trauma made the odds of psychosis 193 times more likely. There is also found to be a cumulative relationship between trauma and psychotic experiences (4). With the causal link between trauma and psychosis now widely recognized, in terms of both acute trauma reactions (5) and childhood, trait-related, trauma reactions (2, 6), the theoretical application of trauma models to psychosis has become an increasingly important guide for hypotheses and research developments.

Cognitive models of trauma and post-traumatic stress disorder (PTSD) (7, 8) illustrate how acute dissociative states can arise in reaction to extreme or adverse experiences. This reaction is thought to involve a de-synchronization or dissociation of processing between the conceptual and perceptual elements of the experience, resulting in unprocessed perceptual memories that lack spatial, temporal, and conceptual context. Moreover, if exposure to such adverse experiences is prolonged or repeated, especially during childhood, dissociative states and processes can become more a part of personality formation, i.e., dissociative traits (9). Both state- and trait-related dissociation can create the potential for de-contextualized memories to be triggered into awareness, experienced by the individual as involuntary perceptual intrusions.

Such “multi-level” trauma processing models have been applied to formulating psychotic symptoms (5, 10–13). According to Morrison et al. (11), unusual perceptual experiences in psychosis, such as hearing voices, may be phenomenologically similar to the perceptual intrusions in PTSD, such as flashbacks; i.e., dissociated and de-contextualized perceptual memories. However, a crucial difference lies in the appraisal of these intrusions, because, in psychosis, the voice or image may not be directly attributed as a component of memory, but rather a novel experience from an alternative source. The lack of contextual information accompanying the perceptual intrusion will increase its vulnerability to conceptual misinterpretation, which, according to cognitive models, is the main factor in the development of psychosis (10, 14). Fowler et al. (12) argue that “problems in contextual processing associated with vulnerability to psychosis may then have the capacity to distort or exaggerate personally significant threat” (p. 116).

### Specific Trauma Pathways to Specific Symptoms?

Bentall et al. (1) suggest that there may be slightly different trauma pathways for different symptoms of psychosis, citing the heterogeneity of psychosis, with evidence of at least three different clusters of symptoms of schizophrenia: positive, negative, and cognitive disorganization (15). However, Bentall et al. (1) go further to argue for specificity of mechanisms between the different psychotic experiences themselves (i.e., within the positive symptom cluster), suggesting that the dissociation pathway (outlined above) may be more relevant to voice-hearing than to paranoia. After reviewing evidence for different trauma types as determinants for voices and paranoia, Bentall et al. (1) argue that childhood sexual abuse is more implicated in the pathway to voice-hearing and that attachment-disrupting events are more implicated in the pathway to paranoia. However, as the authors note, most of the evidence for specific causal pathways to voices and paranoia does not control for the co-occurrence of these positive symptoms, an issue they address in a study using data from the UK 2007 Adult Psychiatric Morbidity Survey (16). After controlling for co-occurring voices and paranoia, they found that childhood sexual abuse was associated with voices, institutional care (an indicator of disrupted attachment) was associated with paranoia, and physical abuse was associated with both voices and paranoia (16).

Bentall et al.'s (1) case for specificity is provided with some support from a replication using a US survey (17) and a prison sample (18). However, closer examination of these studies does not paint a particularly clear picture. For example, Shevlin et al. (18) found that while childhood sexual abuse did show higher odds for hallucinations (odds ratio 2.37) than paranoia (1.20), living in an institution as a child had only slightly higher odds for paranoia (1.49) than hallucinations (1.09). Bullying, a stronger predictor of paranoia (1.99), does not necessarily represent attachment disruption, but rather inter-personal threat and powerlessness (i.e., similar themes to those in sexual abuse). Unwanted sexual attention in prison was also a stronger predictor of paranoia (1.63) than living in an institution.

The specificity picture is further complicated by evidence of sexual abuse pathways to paranoia (19) and attachment disruption pathways to voices (20). In their cognitive-attachment model of voices (CAV), Berry and Bucci (21) propose that a combination of trauma, disorganized attachment, and dissociation are all involved in the pathway to voices. The CAV model is elaborated, along with a thorough review of all the supporting evidence and implications, by Berry et al. (22). Another interesting aspect to this specificity question emerged in a study of mediating mechanisms among 112 participants with distressing psychotic experiences (23). In this study, dissociation was found to mediate the relationship between childhood trauma and voices (as expected) and the relationship between trauma and paranoia (more surprising). This was the first time that the dissociation-paranoia connection had been made in the psychosis literature [earlier research had shown that dissociation predicted hallucinations and not delusions; (24)]. Their interpretation of this result was to suggest that perceptual anomalies in dissociation may also contribute to the formation of paranoid beliefs, and they recommended future research into threat processing mechanisms in relation to both dissociation and paranoia (23). In a recent commentary, Dorahy and Green (25) suggest that the reason this connection was not made before was due to some divergence in the aims of dissociation and psychosis research. They present studies that indicate “shared neurobiological processes for dissociation and paranoia, manifesting in both increased salience of internal threat and perception of threat” (p. 295). Hardy (26) also notes that issues of diagnostic classification may have complicated research aims and findings, recommending a transdiagnostic approach for the future understanding of trauma pathways to psychotic experiences. Another consideration for future research would be to examine specificity pathways using Holmes et al.'s (27) proposed distinction between two types of dissociation (detachment and compartmentalization).

In summary, Bentall et al.'s (1) call for specificity is well-founded, certainly when it comes to differentiating between positive symptoms, negative symptoms and thought disorder. However, within the psychotic (“altered reality”) domain of positive symptoms, there is evidence to suggest roles of both dissociative and attachment mechanisms along the pathways to both voices [dissociative (28); attachment (20)] and paranoia [dissociative (23); attachment (29)]. There is also evidence for more global, generalized effects of childhood adversities on different symptoms (30), which is contrary to the specificity model. Perhaps, therefore, when it comes to psychotic experiences, instead of discounting mechanisms for the sake of specificity, it might be helpful for researchers to consider multiple interacting-mechanism models, e.g., in the vein of CAV (22), and to investigate the relative contributions of each of these processes to the interactions.

One potential area of overlap with trauma pathways (e.g., around social adversity risk factors and attachment processes) is social pathways to psychosis, and these are reviewed below.

## Social Pathways to Psychosis

### Social Factors in Risk of Psychosis

There is considerable evidence that adverse social experiences are risk factors for psychosis. However, broader interpersonal experience, beyond traditional definitions of “trauma,” which can also increase the risk of psychosis, includes inner city living (31), isolation/loneliness (32), discrimination (33), and migration (34). Each will be outlined in turn.

Inner city (urban) living is believed to create a risk of psychosis due to the lack of social cohesion and neighborhood trust typical of urban environments, combined with the higher incidence of crime (35); factors which lead to people feeling socially unsafe. Newbury et al. (35) found that adolescents raised in urban neighborhoods, compared to rural neighborhoods, were significantly more likely to have psychotic experiences (odds ratio 1.67). Interestingly, when Kirkbride et al. (36) studied the notion of social capital, i.e., a measure of embedded community characteristics that provide support, they found a *U*-shaped relationship, whereby both low and high rates of social capital were associated with psychosis. They suggested that while social capital may have benefits, e.g., in buffering social stress, there may also be costs to individuals who are excluded from the social capital that is available (36).

In a systematic review of loneliness in psychosis, Lim et al. (37) define loneliness as when “one perceives their relationships to be inadequate to meet their need for belonging” (p. 221). Their review highlights evidence for a significant positive relationship between loneliness and psychotic symptoms (32); however they suggest that whether this is a causal relationship is unclear due to lack of quality studies. One causal pathway from social isolation to psychosis has been proposed by Hoffman (38) in the *social deafferentation hypothesis*, which suggests that when the social brain is deprived of input (through isolation), the neural networks for processing the social intentions, actions etc. of others produce “spurious” social meaning in the form of voices and delusions. However, there is no empirical evidence to support the causal claims, let alone the precise mechanisms of causation, and further research is needed to support recent calls for psychosocial interventions to target loneliness in psychosis (37).

Perceived discrimination has been demonstrated as a risk factor for psychosis (39, 40), and there have also been suggestions that perceived discrimination could be a major contributory mechanism in the well-established observation of higher psychosis rates in ethnic minority populations (33). In a clinical high-risk group, Stowkowy et al. (41) found that perceived discrimination was an even stronger predictor of later conversion to psychosis than childhood trauma and bullying. In cognitive models, it is suggested that experiences of discrimination and/or perceived discrimination have an influence on the formation of beliefs and schemas, resulting in a paranoid attribution style (33). In evolutionary psychology accounts, discrimination and marginalization experiences are likely to activate social rank mechanisms (42), which is addressed in the following section.

The consistent evidence for higher incidence of psychosis among minority ethnic populations has not only increased the focus on discrimination experiences, but also on migration more generally. In a review of the literature, Morgan et al. (43) identified a number of social variables, in addition to perceived discrimination, that may contribute to the relationship between migration and psychosis. These include childhood factors, such as separation from a parent at a young age, as well as adult social disadvantage factors, such as being unemployed, living alone, being single, poorly educated, and having a limited social network. Other potential factors include the greater likelihood of migrants to live in urban areas (see above), as well as having exposure to the inevitable stressors of transitioning between countries, such as “unfamiliar cultural practices and beliefs, different climate and environment, challenging interactions with government institutions, and for some a new language” [p. 658, Morgan et al. (43)].

In addition to these individual social risk factors, there is also evidence for an accumulation of risk when multiple social factors are combined (44), as well as for the impact of accumulating social risk on gene-environment interactions (45). Environment-gene interactions are now often studied within the relatively new science of epigenetics [for a review of epigenetics in psychosis see (46)]. Although beyond the scope of this paper, it is likely that our future understanding of social pathways to psychosis will involve a better understanding of how social and environmental factors influence gene activity. This will also help us to develop more integrated bio-psycho-social models of psychosis pathways.

### Social Context of Anomalous Experiences

Research on the effects of social factors on anomalous experiences is based on the assumption that these psychotic-like anomalous experiences exist on a continuum throughout the population (47), and that while some people with these experiences may transition into developing a psychotic disorder, the majority will continue with their lives with no detrimental effect to well-being. It is therefore important to establish which factors are involved in transitioning to psychosis, and which factors may be protective. One research strategy has been to compare clinical and non-clinical groups with anomalous experiences (48–50). In these two groups, who do not differ in prevalence of childhood trauma (51), a number of cognitive factors have been identified that likely increase the risk of developing psychosis and a need for care, for example, where experiences are appraised as more threatening and less controllable (50).

In one of the earliest studies comparing clinical and non-clinical groups, Jackson and Fulford (52) suggested that social feedback is likely to be an important factor in determining how an anomalous experience becomes evaluated and embedded in a person's life. They describe the case of “Sara” who firstly described her experiences as terrifying before she received validation from a priest. The authors suggest that the kinds of social responses people receive from others have an important bearing on the consequences of that experience (possibly more so from an authority figure like a priest or even a doctor) (52). In a study using interpretive phenomenological analysis (IPA), the inter-personal context of anomalous experience was identified as being a key factor differentiating clinical from non-clinical groups (49). We found that more clinical than non-clinical participants had received invalidating social responses about their experiences. A quote from “Daniel,” a non-clinical participant, illustrated the subjective importance to him of a validating social experience: “I needed affirmation, that's what I needed… to help me contextualize it and make sense of it… I suppose I did need kind of affirmation from other people that it was all ok” (p. 46).

Other studies have emphasized the helpfulness of socially validating contexts. For instance, Brett et al. (53) identified perceived social support/understanding as a predictor of lower distress among people with anomalous experiences, and Powers et al. (54) found that “psychics” who hear voices report more positive social reactions from their peers than do clinically diagnosed participants who hear voices. Among people with a diagnosed psychotic disorder, social support is also found to be a protective factor in the course of psychosis; for example, higher levels of social support were associated with lower psychotic symptoms and fewer hospitalisations over the 3 years after a first episode psychosis (55).

An important finding of Brett et al. (48), and later replicated (53), is the emergence of spiritual appraisals as a protective factor. In a study of the socio-demographic characteristics of groups with anomalous experiences, Peters et al. (51) reported that a greater proportion of non-clinical participants (over 90%), compared to control and clinical participants, described themselves as spiritual. From a cognitive perspective, the interpretation is that spiritual appraisals may themselves be beneficial (i.e., less distressing, or less threatening ways of making sense of an anomalous experience); however, from a social perspective, the interpretation differs; that spirituality and spiritual appraisal may be indicative of people's access to accepting, validating social groups, with the protective benefits coming more from the social experience of feeling safe, connected, and supported. Most likely, perhaps, it is a combination of the two, but it would be helpful to disentangle these social and cognitive mechanisms. This also applies to the interpretation, and potential re-interpretation of some of the historical research in this field. Traditionally, cognitive models of psychosis have focused on how adverse social experiences contribute to maladaptive cognitive appraisals of experiences; for example, Garety et al. (14) state that “it seems likely that social marginalization, difficult or traumatic experiences or unsupportive family environments contribute to the development of negative schemas” (p. 192). As seen in the above section, and will be seen in the next sections, there are other ways of conceptualizing and interpreting these pathways, e.g., in terms of dissociation, attachment, and social rank processes.

### Social Rank Mechanisms

According to social rank theory, there are information processing systems that evolved specifically for monitoring dominant-subordinate social roles and social threat (56). These systems are primed to detect information about the whereabouts and the intent of others, for example, whether their intent is friendly or hostile. When a person has experienced significant hostility or threat from a powerful other, the social rank systems will be attuned toward threat and can be easily activated (57). Social rank theory has been applied to understanding voice-hearing (58), voice content (59), paranoia (60), and bi-polar disorder (61). A slightly different, but highly relevant model, has also been applied to understanding the more global concept of “schizophrenia,” namely the social defeat hypothesis (62).

The social defeat hypothesis of schizophrenia (62) is based on the observation that social defeat may be a common mechanism underlying many of the major schizophrenia risk factors. The authors argue that social defeat processes can explain associations between schizophrenia with urban living, migration, low IQ, childhood trauma, and use of illicit drugs. Crucially, Selten et al. (62) also link social defeat mechanisms with the well-established neuro-chemical substrates of psychosis, namely dopamine over-activity in the mesolimbic pathway. For a narrative review of the biological arguments linking social defeat to psychosis see Selten et al. (63). In a large scale study of the 6,646 participants in the Netherlands Mental Health Survey (NEMISIS-2), social defeat was found to be a mediator in the relationship between childhood trauma and psychosis (64). This provided strong support for the social defeat hypothesis, but with cross-sectional data only, the authors were unable to conclude that social defeat causes psychosis. In a later study, Seo and Choi (60) also tested a mediation model of social defeat, but this time, in the relationship between childhood trauma and paranoia. Their sample was 199 Korean psychiatric patients, and they used structural equation modeling to demonstrate a pathway from childhood trauma to paranoia, through social defeat.

Social defeat and social rank are slightly different, but related, concepts. Social ranking is an evolutionary psychology concept, referring to the way humans have evolved to co-ordinate social (group) living, whereby low rank members are subordinate to high rank members, and that group cohesion (and self-preservation) is achieved by “hard-wired” patterns of mental organization, perception, feeling, behavior, etc., called social “mentalities” (65). So, for low rank members this involves perceiving oneself as inferior, thinking that others look down on them, and behaving submissively. Social defeat, on the other hand is the (current) experience of one being put down by a dominant other. Therefore, perceptions of social defeat may be highly associated with perceptions of low social rank (66). Also, social defeat experiences, particularly those which are repeated or prolonged, are likely to activate and *attune* social rank patterns. Other experiences that might activate social rank “mentalities” include entrapment (i.e., being unable to escape an uncontrollable situation), being criticized, bullied, shamed, or disempowered, as well as some internal experiences (in the self-to-self relationship) such as self-criticism and self-stigma.

The observations and evidence in support of the social defeat hypothesis of schizophrenia are therefore relevant in the application of social rank theory in psychosis. What social rank theory adds, however, is an understanding of the evolutionary context and function of these mechanisms. It also provides a framework for understanding the contribution of the self-to-self relationship of people with psychosis, and, for voice-hearers, the relationship people have with their voices. For example, as suggested by Heriot-Maitland et al. (57), social rank theory might help us understand why voice-hearing can involve “an internal ‘playing-out' of both the hostile-dominant and the (reciprocal) threatened-subordinate social roles” (p. 152). In other words, both the high rank and the low rank poles of the social rank system are activated in the relationship between voice and voice-hearer.

In the past, social rank processes have most commonly been connected to depression (56, 67) and therefore applications of social rank theory in psychosis have often focused on affective pathways, with social rank being implicated in negative symptoms and depression outcomes in psychosis. For example, in a large sample of 2,350 online participants, Jaya et al. (68) analyzed the effects of three mediators (social rank, negative schemas, and loneliness) on the relationship between social adversity and psychosis (positive and negative symptoms). Low social rank, as measured by the Social Comparison Scale (SCS) (69), was found to be a significant mediator in the pathway to both negative symptoms and depression, but not in the pathway to positive symptoms. In their study, only negative schema mediated the relationship between social adversity and positive symptoms, and their results suggest that social rank may be more relevant to affective and depressive aspects of psychosis. However, other studies by Birchwood's group have highlighted the role of social rank mechanisms in voice-hearing (a positive symptom), and how voice relationships are influenced by perceptions of social rank in other relationships (42, 70–72). In a review of this work, Birchwood et al. (58) not only provide evidence that voice-hearing reflects a perception of low social rank, but also propose the applicability to other symptoms, including delusions and negative symptoms, as well as to broader aspects of the psychosis experience:

“*Recent research has given strong support to the application of social rank theory to psychosis and shows that apparently disparate aspects of the psychotic experience—from voices to family relationships to diagnosis and hospitalization—are all facets of the same process. This process involves a catastrophic loss of status in social rank terms, resulting in involuntary subordination, humiliation, and loss of self-esteem, and entrapment by powerful others. This can lead to anxiety, depression, and relapse in an ever-worsening decline in social status.”* [p. 144, Birchwood et al. (58)]

Another important social rank variable is shame (73–78). Shame, and a host of related concepts, such as internalized stigma (79, 80) and self-stigma (81, 82) all reflect social rank patterns, in either self-to-other or self-to-self relationships, and can all result from experiences of being victimized or stigmatized. The experience of being seen as negative or inferior in the eyes of others (external shame) or perceiving oneself as inferior (internal shame) can be understood in terms of “social rank threats,” in that they are threats to one's social self, and similar to social defeat experiences, can activate and attune social rank patterns.

A recent systemic review of shame and psychosis (83) identified 20 eligible papers with studies of clinical (*n* = 8), non-clinical (*n* = 8), and mixed clinical-non-clinical samples (*n* = 4). In summary, they found evidence of a moderate-to-strong relationship in studies of shame and psychosis (clinical) and psychotic-like experiences (non-clinical). In relation to symptom-specific associations, they found that more studies showed positive associations between shame and paranoia than voices, and, importantly, one study also showed that shame had an amplifying effect on the relationship between stressful life events and paranoia (84). A limitation was that all 20 studies included in this review were cross-sectional, and so future longitudinal research is needed in order to identity the directionality of these relationships.

In summary, there is a considerable literature implicating social rank mechanisms in psychosis. Although different terminology has been adopted by different researchers (e.g., social defeat, shame, internalized stigma, etc.), there are clear similarities at their core in terms of the activation of dominant-subordinate social rank mechanisms. Taking the broader literature together, therefore, social rank threats and mechanisms have been linked to many different aspects of psychosis: risk factors (social adversity risks and interpersonal trauma); depression in psychosis, and positive symptoms (paranoia and voice-hearing). Shame has also been linked to the expressed emotion pathway (85, 86), and as mentioned above, the biology of social defeat has been connected to the biological (e.g., dopaminergic) profile of psychosis. Finally, as Birchwood et al. (58) highlight, social rank processes are important in understanding the wider social context and consequences of psychosis. However, as much of the research is cross-sectional, further studies are needed to test these processes with longitudinal designs.

### Attachment Mechanisms

Like social rank theory, attachment theory is an evolutionary model that explains the organization of social interactions. Where social rank explains how social groups organize themselves hierarchically, attachment explains how relationships are organized for caring and nurturing, ultimately in the interests of survival. According to attachment theory, human infants have a biological (“built in”) drive to seek closeness to a protective caregiver, and to feel safe / secure within this affiliative bond (87). Securely attached infants use this relationship with the caregiver as both a *safe haven* (to calm distress) and *secure base* (from which to explore). However, in some inter-personal developmental contexts, these attachment relationships may not be possible, and so instead people develop what are called “insecure” attachment patterns and styles. These patterns have been delineated in subgroups, e.g., anxious, avoidant, and disorganized attachment style, and each pattern has an important bearing on the growing child's social functioning and emotion regulation.

A review of the literature on attachment and psychosis by Berry et al. (88) highlighted the high rates of insecure attachment among people with psychosis and suggested a number of ways in which attachment ideas could contribute to psychological understandings and treatments of psychosis. A later systematic review by Gumley et al. (89) identified good evidence to support the relationship between avoidant attachment and both positive and negative symptoms of psychosis. They also found modest evidence to support a relationship between anxious attachment and positive symptoms. In their review, they also identified two studies that linked attachment insecurity to trauma, and particularly interpersonal traumatic events (90, 91). Recently, Lavin et al. (92) reviewed the literature specially looking at the relationship between insecure attachment and paranoia in psychosis. They found significant associations in 11 of 12 studies, and a pattern within these results whereby anxious attachment style was more strongly associated with paranoia than avoidant attachment style.

The theoretical literature on attachment and psychosis has mostly centered around disorganized attachment, which is an insecure pattern that often results from childhood trauma, and stems from a conflict between the attachment system and the threat-protection system (93). In their theoretical account, Liotti and Gumley (94) highlight the roles of attachment disorganization, trauma and dissociation in psychosis, suggesting that attachment disorganization has multiple influences on the pathway to psychosis, specifically through (i) hindering affect regulation, (ii) facilitating dissociative responses to trauma, and (iii) creating difficulties with mentalising. The dissociation and mentalising pathways also have indirect contributions to affect regulation. For a review of the evidence and mechanisms of mentalising deficits on the psychosis continuum, see Debbane et al. (95).

In summary, the attachment literature points toward disorganized attachment and dissociation as being implicated in the eliciting of psychotic experiences (e.g., voices), toward insecure attachment being implicated in the appraisal of experiences (21), and toward insecure anxious attachment specifically in more paranoid thinking/appraisal styles (92). Again, the further testing of these relationships in longitudinal studies is needed to evidence causality.

## Where the Two Paths Meet: Interaction of Trauma and Social Pathways

The review of pathways to psychosis in the previous section has identified a number of potential mechanisms, some traumatic/dissociative, and others social/inter-personal. It also identified areas where their empirical and theoretical strands overlap; for example, the case of inter-personal trauma, which is linked to multiple different pathways, both dissociative and social. It may be that each mechanism could be activated by different (independent) determinants, or, in the case of inter-personal trauma, by the same determinant. There is also a chance that one or more mechanisms could interact, and potentially become determinants of each other, which is the topic of this section. Already noted has been one important model of integrated mechanisms, namely CAV (22), which describes the dissociation and attachment interactions in voice-hearing. In another integrated model, although not in the psychosis field, Sloman and Taylor (96) outline an evolutionary account of childhood maltreatment in terms of the interaction between attachment and social rank systems. The focus will now turn to the area of interaction between dissociation and social rank.

### The Traumatic/Dissociative Properties of Shame

As social animals, some of the most important threats to humans are those that operate in the social realm, for example, threats of social devaluation, rejection, and isolation. As Gilbert (97) points out, while the threat for most animals is aggression, for humans it is “more commonly related to loss of acceptance and approval” (p. 175). In the psychosis literature, previous studies of shame (a socially-conscious emotion) have typically focused on the relationship between shame and emotional symptoms in the context of psychosis [e.g., anxiety (73) and depression (75–77)]. Shame has also been researched as a consequence of psychosis (81), as well as potentially having a role in post-psychotic trauma (74). Less attention, however, has been paid to shame as a potential causal mechanism in dissociative and psychotic experiences themselves. This may partly be due to shame not traditionally being regarded in “threat” terms, with e.g., traumatic-like, perhaps dissociative, properties of its own. However, what social rank theory offers is an understanding of shame within its evolutionary context, and with links to survival strategies. There are strong theoretical grounds to examine shame through the lens of threat and evolved threat-protection mechanisms. From this angle, social experiences, such as stigma, shame, and other threats to social self (98) can be investigated for links to traumatic, dissociative processes, and subsequently as potential causal mechanisms in psychosis.

There is evidence for a relationship between shame and dissociation; for example Talbot et al. (99) found that levels of shame were positively associated with levels of dissociation, and that the strength of this relationship was greater among participants with childhood sexual abuse. Dorahy et al. (100) tested a causal relationship between shame and dissociation. They gave students (*n* = 78) both shame-inducing and neutral scripts, and while scripts were being listened to and repeated, participants were assessed on measures of shame (e.g., internal, external, eye gaze) and dissociation. They found that in the presence of shame activation, dissociative states increased. These findings give evidence for threat- or traumatic-like properties of shame (100), which are therefore of relevance to psychosis research. In another series of studies, Matos et al. provided evidence for traumatic characteristics of shame memories (101–103), and also found that the more traumatic a shame memory, and the more central it is to one's identity, the more it predicts paranoia in the general population (103). Again, these studies suggest a more causal role for shame in dissociative phenomena, as well as in psychotic-like phenomena.

Other studies have investigated interactions between dissociation and threat activation. Although these studies have not looked specifically at shame, they may still have relevance in terms of understanding the consequences when dissociative traits and current threat interact. For example, Holmes et al. (104) researched the interaction of dissociation with a current threat stimuli (of watching an aversive film) in producing intrusions among non-clinical participants. They measured the intrusions experienced by participants following exposure to the film under experimental conditions that manipulated the participants' conceptual processing of threat stimuli, relative to perceptual processing. They found that both decreased conceptual processing of threat (an experimentally simulated trait dissociation), and spontaneous increases in state dissociation, led to increased intrusions of threat stimuli after the film. In another study, Marks et al. (105) showed that healthy individuals with psychotic-like experiences had lower level trait conceptual integration, and more intrusions, following an aversive film than controls. Although these studies did not specifically look at shame, they do pose interesting research questions for dissociation-shame interactions. For example, if, as suggested, shame is understood to operate as a threat stimulus (social threat), might we also expect dissociation-shame interactions to lead to increased intrusions?

### Summary of the Pathways and Their Areas of Interaction

In pathways to psychosis, there is evidence for mechanisms of (i) dissociation, (ii) attachment disruption, and (iii) social rank/shame. Inter-personal trauma has relevance as a determinant for all three mechanisms, as it has both the traumatic/dissociative aspects, as well as the social/inter-personal aspects. There are a range of other potential determinants for each mechanism, for example, those that potentially lead to (i) dissociation (e.g., non-personal trauma, drug-use, existential crisis/impasse); (ii) attachment disruption (e.g., parental mental health, separation, institutional living, neglect); and (iii) social rank/shame (e.g., discrimination, bullying, criticism, expressed emotion, and stigmatization). There are also potential ways in which these mechanisms could interact, essentially becoming determinants of each other. This could be, for example, through the influence of either attachment (22, 106) or shame (100), or both, on dissociation.

**Figure 1:**
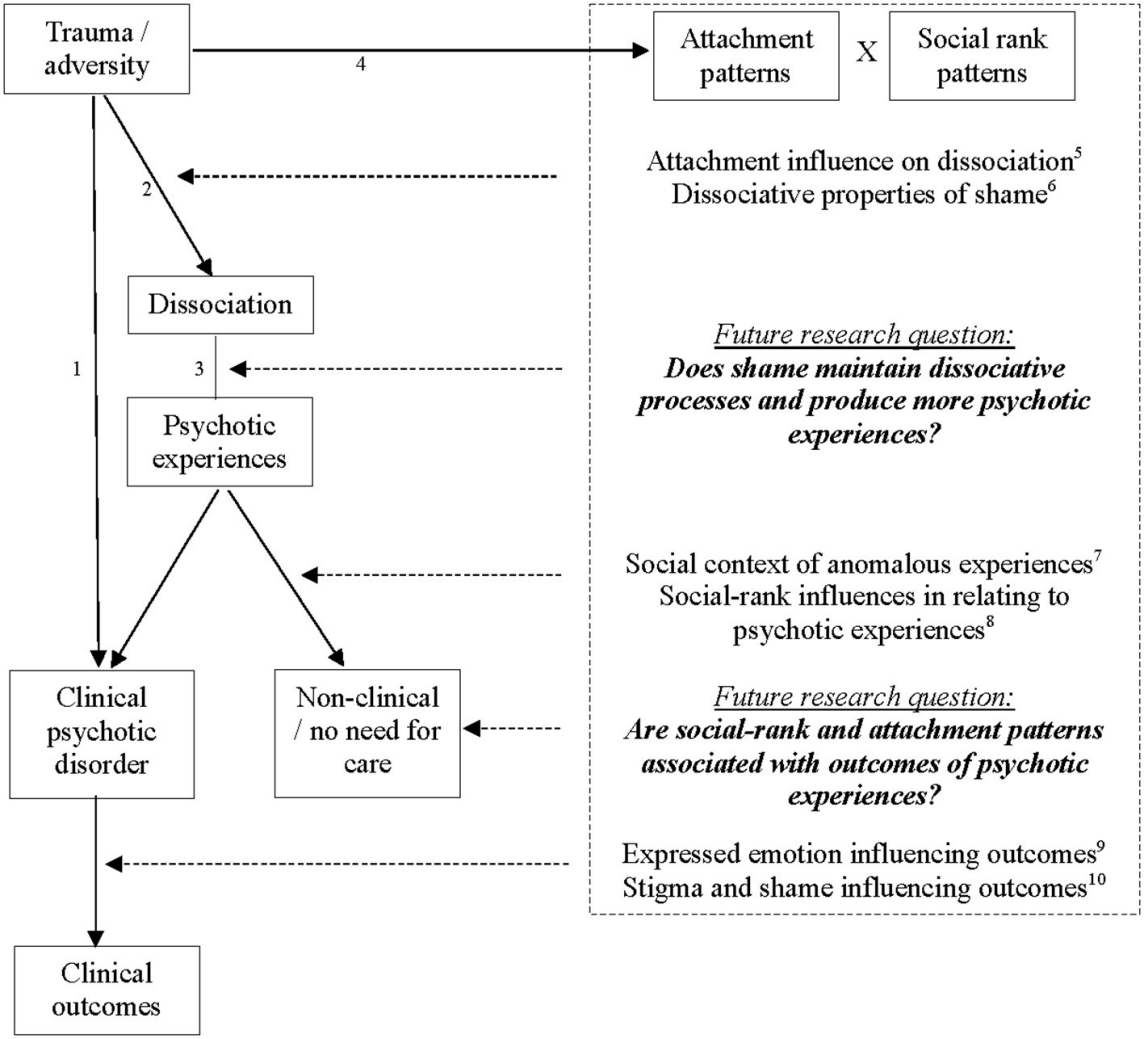
Social influences on the trauma pathway to psychosis. ^1^Trauma relationship with psychosis (1); ^2^Trauma relationship with dissociation (28, 107); ^3^Dissociation relationship with psychotic experiences (108, 109); ^4^Trauma relationship with attachment (20, 23) and social rank/shame (60, 64, 99) in psychosis; ^5^Liotti (106) and Berry et al. (22); ^6^Matos and Pinto-Gouveia (101) and Dorahy et al. (100); ^7^Heriot-Maitland et al. (49) and Brett et al. (53); ^8^Gilbert et al. (71) and Birchwood et al. (72); ^9^Bebbington and Kuipers (110) and Onwumere et al. (111); ^10^Turner et al. (74), Upthegrove et al. (75), and Keen et al. (77).

Figure 1 provides a diagrammatic representation of the main areas reviewed in this article, with a particular aim of illustrating the areas of interaction, i.e., the social influences (attachment or social rank) on different parts of the trauma pathway to psychosis. The diagram is not intended to show the whole picture, but to illustrate where this review sits in relation to the literature, with the suggestions for future research questions highlighted in bold.

## Conclusion

This review has highlighted ways in which psychosis modeling might be fine-tuned going forward; for instance, it supports the approach taken by Berry et al. (22) in developing integrated, interacting mechanism models of pathways to psychosis. It also emphasizes the need to develop models of social influences in *producing* psychotic experiences themselves, rather than what has traditionally been done, which is to model social influences on processes that occur *after* psychotic experiences, e.g., social influences on their cognitive appraisals, the social consequences of psychosis in terms of stigma, shame, and internalized stigma, and the social influences on psychosis relapse. A specific theoretical recommendation is for future modeling of three-way interacting mechanisms (dissociation × attachment × social rank) in pathways to psychosis, which, in turn, gives rise to a number of interesting research hypotheses and questions about how these mechanisms interact—for example, (a) do social rank and attachment mechanisms influence dissociation? (b) do social rank mechanisms have an *additional* influence to that of attachment on dissociation? and (c) how do these three-way interactions influence trauma pathways to voices (specifically), paranoia (specifically), and psychotic experiences more generally? More research in this area could inform future directions in how to help people with psychosis, not only in terms of designing therapies that specifically target these mechanisms, but also in promoting social experiences as vehicles for recovery and change across multiple levels—from family and friends, to peer-support and groups, to shaping the wider inter-personal culture in services.

## Author Contributions

CH-M wrote the first draft of the manuscript. All authors contributed to manuscript revisions, read, and approved the submitted version.

## Funding

This work was supported by a Medical Research Council Fellowship (CH-M, grant number MR/L01677X/1).

## Conflict of Interest

The authors declare that the research was conducted in the absence of any commercial or financial relationships that could be construed as a potential conflict of interest.

## Publisher's Note

All claims expressed in this article are solely those of the authors and do not necessarily represent those of their affiliated organizations, or those of the publisher, the editors and the reviewers. Any product that may be evaluated in this article, or claim that may be made by its manufacturer, is not guaranteed or endorsed by the publisher.
